# MicroRNA-340 inhibits the proliferation and promotes the apoptosis of colon cancer cells by modulating REV3L

**DOI:** 10.18632/oncotarget.23703

**Published:** 2017-12-26

**Authors:** Roshini Arivazhagan, Jaesuk Lee, Delger Bayarsaikhan, Peter Kwak, Myeongjoo Son, Kyunghee Byun, Ghasem Hosseini Salekdeh, Bonghee Lee

**Affiliations:** ^1^ Center for Genomics and Proteomics, Lee Gil Ya Cancer and Diabetes Institute, Gachon University, Incheon, Republic of Korea; ^2^ Department of Anatomy and Cell Biology, Gachon University Graduate School of Medicine, Incheon, Republic of Korea; ^3^ Department of Molecular Systems Biology, Cell Science Research Center, Royan Institute for Stem Cell Biology and Technology, ACECR, Tehran, Iran; ^4^ Department of Molecular Sciences, Macquarie University Sydney, New South Wales, Australia

**Keywords:** REV3L, mislocalization, interactions, colon cancer, miR-340

## Abstract

DNA Directed Polymerase Zeta Catalytic Subunit (REV3L) has recently emerged as an important oncogene. Although the expressions of REV3L are similar in normal and cancer cells, several mutations in REV3L have been shown to play important roles in cancer. These mutations cause proteins misfolding and mislocalization, which in turn alters their interactions and biological functions. miRNAs play important regulatory roles during the progression and metastasis of several human cancers. This study was undertaken to determine how changes in the location and interactions of REV3L regulate colon cancer progression. REV3L protein mislocalization confirmed from the immunostaining results and the known interactions of REV3L was found to be broken as seen from the PLA assay results. The mislocalized REV3L might interact with new proteins partners in the cytoplasm which in turn may play role in regulating colon cancer progression. hsa-miR-340 (miR-340), a microRNA down-regulated in colon cancer, was used to bind to and downregulate REV3L, and found to control the proliferation and induce the apoptosis of colon cancer cells (HCT-116 and DLD-1) via the MAPK pathway. Furthermore, this down-regulation of REV3L also diminished colon cancer cell migration, and down-regulated MMP-2 and MMP-9. Combined treatment of colon cancer cells with miR-340 and 5-FU enhanced the inhibitory effects of 5-FU. In addition, *in vivo* experiments conducted on nude mice revealed tumor sizes were smaller in a HCT-116-miR-340 injected group than in a HCT-116-pCMV injected group. Our findings suggest mutations in REV3L causes protein mislocalization to the cytoplasm, breaking its interaction and is believed to form new protein interactions in cytoplasm contributing to colon cancer progression. Accordingly, microRNA-340 appears to be a good candidate for colon cancer therapy.

## INTRODUCTION

Colorectal cancer (CRC) is one of the most common types of cancer, and although its five-year survival rate has increased, it remains the second most common cause of cancer-related death. The major cause of CRC is the accumulation of mutations resulting in uncontrolled cellular proliferation and survival, and the majority of these genetic changes affect members of the Wnt/β-catenin signaling pathway and cause the loss of p53 [1–4]. Due to increases in drug resistance and recurrence rates, the discovery of an effective treatment for CRC remains a daunting challenge [5]. Many of the chemotherapeutics employed in cancer therapy are severely limited by their adverse side effects, and hence, it is important to find improved means for treating CRC that minimize side effects and recurrence rates.

Translesion DNA synthesis (TLS) is a process whereby mutations that occur during DNA replication are bypassed, allowing cell to complete the genome replication by recruiting specialized DNA polymerases at the replication forks [6–8]. Furthermore, these polymerases play roles in spontaneous and DNA damage-induced mutagenesis, and thus, contribute to the malignant transformations of cells [1, 9]. REV3L, the catalytic subunit of POLζ, plays a major role in the DNA damage tolerance mechanism of TLS [10, 11]. REV7, REV1, and MAD2L2 interact with POLζ, and REV3L has been reported to cause drug resistance in several types of cancers [9, 12, 13]. In particular, one study showed that silencing REV3L caused persistent DNA damage and growth arrest in cancer cells [14, 15].

Although REV3L is not overexpressed in several cancer types, it plays an important role in tumorigenesis. In fact, it has been reported to affect the survival rate and prognosis in different types of cancers like cervical cancer, glioma etc. [2, 5, 16, 17]. DNA Polymerase ζ (POLζ) contributes to drug resistance by allowing cancer cells to survive despite treatment withmany types of DNA damaging agents [18–23]. Protein mislocation, that is, a change in the subcellular location of a protein influences cellular function and the control of disease, as interacting partners are no longer availableand protein functions are altered. These mislocations are usuallycaused by genetic mutations that cause fold level expressional changes and alter protein functions. Reports on genetic mutations in REV3L are consistent in several cancer types, including lung and breast cancer, and mutations in its UTR region prevent microRNAs (miRNAs) from silencing REV3L in cancer [24–28]. miRNAs are 18-25 nucleotide, noncoding RNAs that base pair to complementary sequences in the 3’ untranslated region (UTR) of target mRNA, and thus, blockgene expression. Thousands of miRNAs have been identified in humans and these regulate ∼30% of genes [29, 30]. Furthermore, miRNAs have been associated with many diseases, including cancers, in which they may act as tumor suppressors or oncogenes [31–33]. miR-340 acts as a tumor suppressor in glioma, melanoma, hepatocellular carcinoma, in breast and gastric cancer, and others [34–37], and has been shown to disrupt the progressionsofmany cancer types by targeting important oncogenes. However, miR-340 is downregulated in these cancers [38–40].

This study was performed to investigate the changes in the location and interactions of REV3L and the impact in regulating colon cancer progression. Downregulation of REV3L with miR-340 showed to control the colon cancer cell proliferation and induced apoptosis as shown by the cell viability assay, TUNEL assay and colony formation assay. Nude mice model was used to determine the effect of miR-340 mediated blocking of REV3L in colon cancer progression *in vivo*.

## RESULTS

### REV3L was mislocalized to cytoplasm in colon cancer cell lines

**Figure 1:**
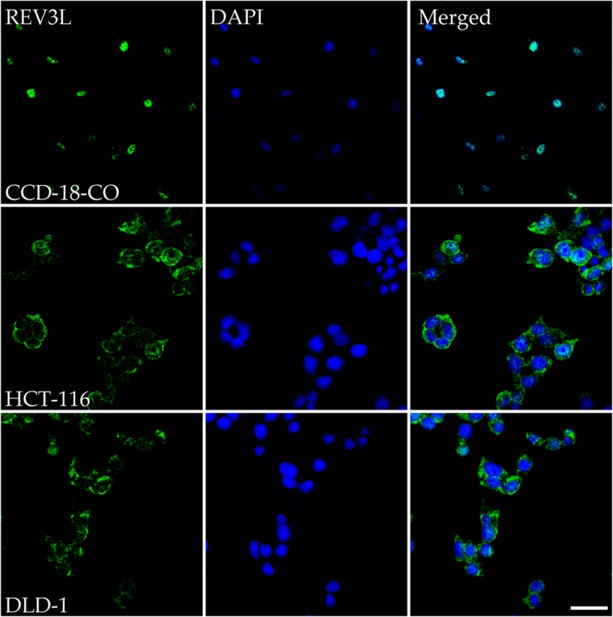
Immunostaining analysis of the location of REV3L in normal colon and colon cancer cell lines Normal colon cell line CCD-18-Co shows REV3L nuclear location, coloncancer cell lines HCT-116 and DLD-1 shows mislocalisation of the protein upon immunostaining with REV3L antibody and counter staining with DAPI. The images were analyzed using an LSM 710 confocal microscope (Carl Zeiss). Scale bar 200μm.

Immunostaining was used to evaluate the subcellular localizations of REV3L in normal and colon cancer cells. Staining results for the normal colon cell line CCD-18Co showed REV3L protein localized in nuclei. On the other hand, immunostaining of REV3L in colon cancer cell lines HCT-116 and DLD-1 revealed its mislocalization to cytoplasm (Figure 1).

### Protein interactions between REV3L, REV1, and MAD2L2 were markedly lower in colon cancer cells than in normal colon cells

**Figure 2:**
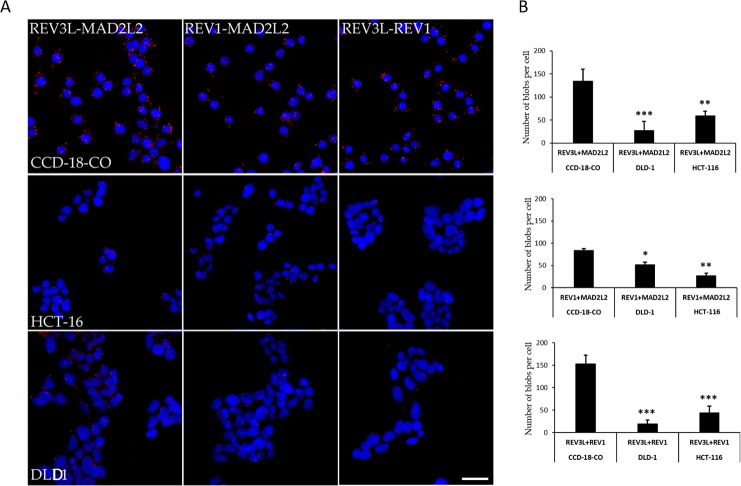
Protein-Protein interaction of REV3L in Normal colon and Colon cancer cell lines **(A)** PLA assay for REV3L interaction with REV1 and MAD2L2 in CCD-18-Co normalcolon and HCT-116, DLD-1 colon cancer cells. The red blobs indicate the protein interactions. The images were analyzed using an LSM 710 confocal microscope (Carl Zeiss). **(B)** The number of interactions between REV3L-REV1, REV3L-MAD2L2, REV1-MAD2L2 in CCD-18-Co normal colon and HCT-116, DLD-1 colon cancer cells plotted as graph. The data are presented as means with SDs for three independent experiments. ^*^p<0.05; ^**^p<0.01; ^***^p<0.001. Scale bar 20μm.

A proximity ligation assay (PLA) was used to investigate interactions between REV3L, REV1, and MAD2L2 in normal (CCD-18Co) and colon cancer cells. Interactions between REV3L and REV1, REV3L and MAD2L2, and REV1 and MAD2L2 were at 153/cell, 134/cell, and 84/cell, respectively, in normal colon cells. However, the number of interactions between REV3L and REV1, REV3L and MAD2L2, and REV1 and MAD2L2 were reduced to 44/cell, 27/cell, and 26/cell, respectively, in HCT-116 colon cancer cells and to 19.2/cell, 59/cell, the 51/cell in DLD-1 colon cancer cells. Numbers of interactions (visualized as red blobs) were counted and plotted on a graph (Figure 2A, 2B). These results suggested that the mislocalization of REV3L in colon cancer disrupts interactions between REV1 and MAD2L2, which with REV3L form Polymerase zeta complex, which is known to play a major role in DNA damage repair.

### miR-340 expression in colon cancer was inversely related to REV3L mRNA expression

**Figure 3:**
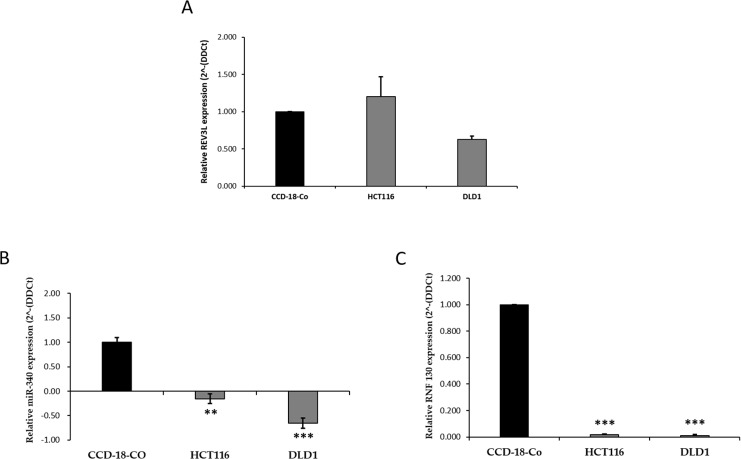
mRNA expression pattern of REV3L, miR-340 and RNF-130 in normal colon and colon cancer cell lines **(A)** mRNA expression levels of REV3L in CCD-18-Co normal coloncell line and HCT-116, DLD-1 colon cancer cells. Cell lines were analyzed for REV3L mRNA levels by qPCR. GAPDH gene was used as endogenous control. **(B)** miR-340 expression levels were quantified in CCD-18-Co normal colon cell line and HCT-116, DLD-1 colon cancer cell lines by qPCR. Expression of miR for each colon cancer cell line was compared with the normal cell line CCD-18-Co. U6 expression was used as endogenous control. **(C)** mRNA expression levels of RNF-130, the intronic region of the gene that codes for miR-340 in CCD-18-Co normal colon cell line and HCT-116, DLD-1 colon cancer cell lines. GAPDH gene was used as endogenous control. Consecutive to miR-340 expression, RNF-130 mRNA expression levels decreases in HCT-116, DLD-1 colon cancer cells compared to CCD-18-Co normal colon cell line. The data are presented as means with SDs for three independent experiments. ^*^p<0.05; ^**^p<0.01; ^***^p<0.001.

We first analyzed the mRNA expression levels of REV3L in the HCT-116 and DLD-1 cells and in CCD-18Co cells by qPCR. The results obtained showed REV3L mRNA levels were similar in the three cell lines (Figure 3A). To identify microRNA that targets and regulates REV3L gene expression, we used prediction algorithms, that is, TargetScan, microRNA.org, miRDB, and Diana microT. All four databases showed that hsa-miR-340 targets REV3L. The basal expression levels of hsa-miR-340 were found to be lower in HCT-116 and DLD-1 cells than in CCD-18Co cells by qPCR (Figure 3B). We then used qPCR to examine the mRNA expression of the gene that code for miR-340 namely, RNF-130, in all three cell lines. As shown in Figure 3C, like miR-340 expression, RNF-130 mRNA expression was lower in colon cancer cells than in the normal colon cell line. These results suggested the location of REV3L protein plays major role in cancer prognosis and that downregulation of miR-340 could have a tumor suppressing function in colon cancer.

### miR-340 regulated REV3L mRNA expression by directly binding to its 3’-UTR region

**Figure 4:**
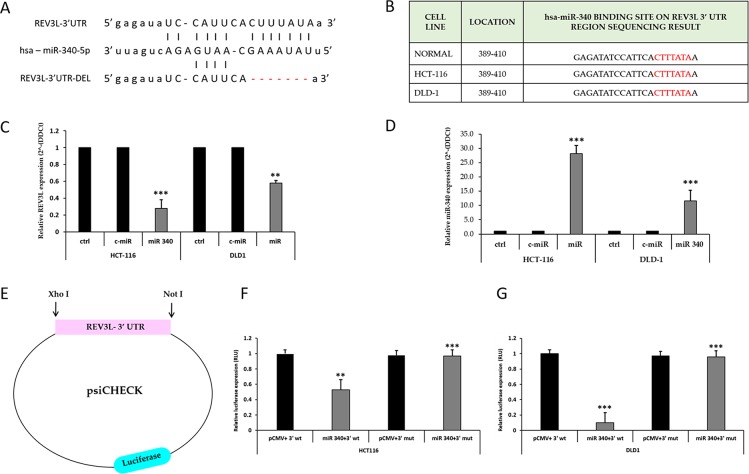
miR-340 targets the 3’-UTR of REV3L **(A)** Binding site of miR-340 to the 3’UTRregion of REV3L. miR-340 binds with 7 complimentary bases at the seed sequence and deletion of the miR-340 binding seed sequence in the 3’UTR region of REV3L. **(B)** Sequencing result of 3’UTR of REV3L in CCD-18-Co normal colon cell line and HCT-116, DLD-1 colon cancer cell lines at miR-340 binding site. **(C)** HCT-116 and DLD-1 colon cancer cells were transfected with 50nM miR-340 mimics. mRNA expression of REV3L upon miR-340 transfection were profiled by qPCR. GAPDH gene was used as endogenous control. **(D)** The relative miR-340 expression level in miR-340 mimic transfected HCT-116 and DLD-1 colon cancer cell lines compared to the not transfected cells and positive control miR transfected cells. U6 expression was used as endogenous control. **(E)** The 3’ UTR of REV3L cloned into psi-CHECK Luciferase vector with XhoI and NotI digestion sites. **(F, G)** Luciferase reporter assay showed that miR-340 directly targets the REV3L 3’-UTR-luciferase reporter (wild type or deleted miR-340 binding seed sequence), in HCT-116 and DLD-1 cells incubated with the miR-340 mimic for 48h before analysis. The firefly luciferase activity of the reporter was normalized to the internal Renilla luciferase activity. The data are presented as means with SDs for three independent experiments. ^*^p<0.05; ^**^p<0.01; ^***^p<0.001.

Next, we sought to determine whether the negative regulatory effect of miR-340 on REV3L expression is mediated by direct binding to predicted sites in the 3’-UTR of REV3L mRNA using four different algorithms (Figure 4A, Supplementary Figure 1). Several reports have described mutations in several miR binding sites in the 3’-UTR region of REV3L. To determine the presence of mutations in the miR-340 binding site on REV3L, we PCR amplified and sequenced the entire 3’-UTR regions of REV3L in CCD-18Co, HCT-116, and DLD-1 cells. Sequencing results confirmed the absence of mutations in the miR-340 binding region of both colon cancer cell lines (Figure 4B). Next, we quantified the mRNA expression of REV3L using qPCR after transfecting HCT-116 and DLD-1 cells with control miR and miR-340 mimics. It was found REV3L mRNAexpression was downregulated and miR-340 expression increased in these cells upon miR-340 transfection, providing additional evidence of miR-340 to REV3L binding (Figure 4C, 4D). We then cloned the 3´UTR region into psi-CHECK2 vector and transfected the resulting plasmid into HCT-116 and DLD-1 colon cancer cells along with pCMV and/or pCMV-miR-340 plasmids. Luciferase activity was significantly reduced in cells transfected with the REV3L 3’-UTR plasmid and miR-340. To confirm binding of miR-340 to the specific binding motif, we also deleted the miR-340 binding site in the 3’-UTR of REV3L (Figure 4E–4G).

### miR-340 overexpression significantly inhibited the proliferation and migration of colon cancer cells and induced apoptosis via the MAPK pathway

**Figure 5:**
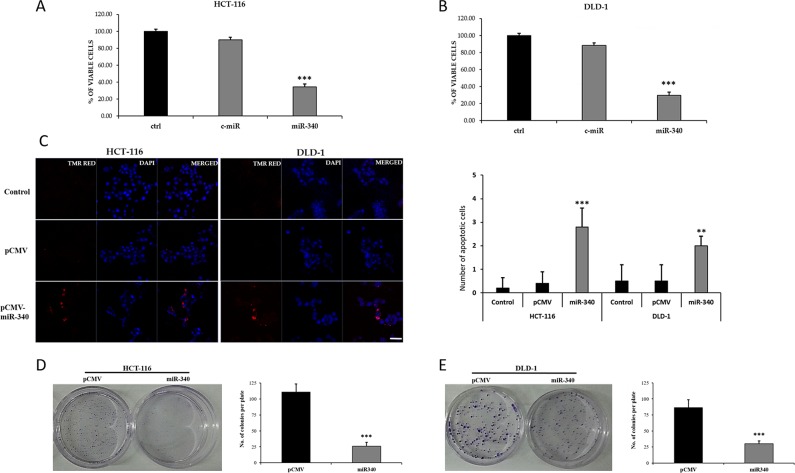
Ectopic expression of miR-340 suppresses colon cancer cell proliferation and induces apoptosis *in vitro* **(A, B)** The short term effects of ectopic expression of miR-340 on theproliferation of HCT-116 and DLD-1 colon cancer cells examined by cell proliferation assay (MTS assay). Graph represents the percentage of viable cells in control and treated groups. **(C)** Effects of ectopic expression of miR-340 on the apoptosis of HCT-116 and DLD-1 colon cancer cells examined by TUNEL assay. The number of TUNEL positive cells were counted and made as graph. **(D, E)** Effects of overexpression of miR-340 on the colony formation or clonogenic ability of HCT-116 and DLD-1 colon cancer cells. Culture dishes represents the number of colonies formed upon transfection with pCMV and pCMV-miR-340. Graph represents the number of colonies formed in pCMV-miR-340 transfected cells normalized to the number of colonies formed by the pCMV transfected cells. The data are presented as means with SDs for three independent experiments. ^*^p<0.05; ^**^p<0.01; ^***^p<0.001. Scale bar 20μm.

To examine the tumor suppressor roles of miR-340, HCT-116 and DLD-1 colon cancer cells were transfected with control miR and miR-340 mimics, and 48h later, MTS assays were performed to determine of viable cell percentages. It was found in miR-340 overexpressing HCT-116 and DLD-1 cells; number of viable cells were dramatically decreased (Figure 5A, 5B). We then examined the apoptotic role of miR-340 by TUNEL assay. Numbers of TUNEL positive cells were higher in miR-340 transfected HCT-116 and DLD-1 cells than in control miR transfected cells (Figure 5C), suggesting that miR-340 induces colon cancer cell apoptosis.

**Figure 6:**
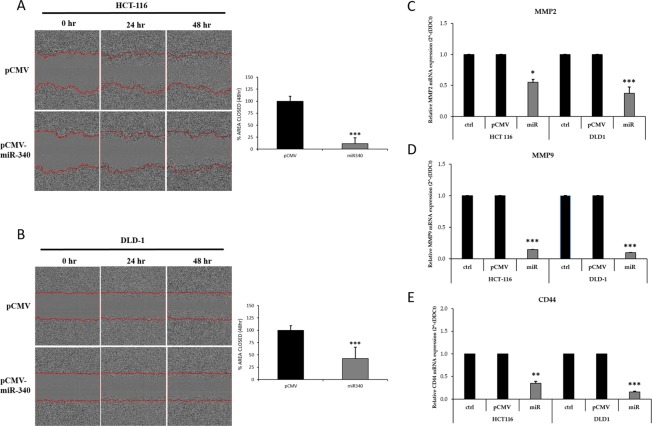
Transfection of colon cancer cells with miR-340 suppresses migration and metastatic properties of cells *in vitro* **(A, B)** HCT-116 and DLD-1 colon cancer celllines that were transfected with pCMV-miR-340, or pCMV were assessed for migration with the wound healing assay. The area of the wound was measured at 0, 24 and 48 h. Graph represents the relative percentages of wound closure of the pCMV and pCMV-miR-340 transfected cells. **(C, D)** HCT-116 and DLD-1 colon cancer cells were transfected with pCMV and pCMV-miR-340 plasmids. mRNA expression of the markers for metastasis MMP2 and MMP-9 upon miR-340 transfection were profiled by qPCR. GAPDH gene was used as endogenous control. The data are presented as means with SDs for three independent experiments. ^*^p<0.05; ^**^p<0.01;^***^p<0.001.

We further examined the effects of miR-340 on colon cancer cell proliferation and clonogenicity using a colony formation assay. pCMV-miR-340-transfected HCT-116 and DLD-1 cells formed fewer and smaller colonies than pCMV transfected controls (Figure 5D, 5E). These results confirmed the growth inhibitory effects of miR-340 on colon cancer cells. The effect of miR-340 on colon cancer cell migration was also evaluated. Scratch area coverages of HCT-116 and DLD-1 cells transfected with pCMV-miR-340 were ∼15% and 35% lower 48h than that of pCMV treated cells (Figure 6A, 6B), indicating miR-340-expression reduces cell motility and migration.

**Figure 7:**
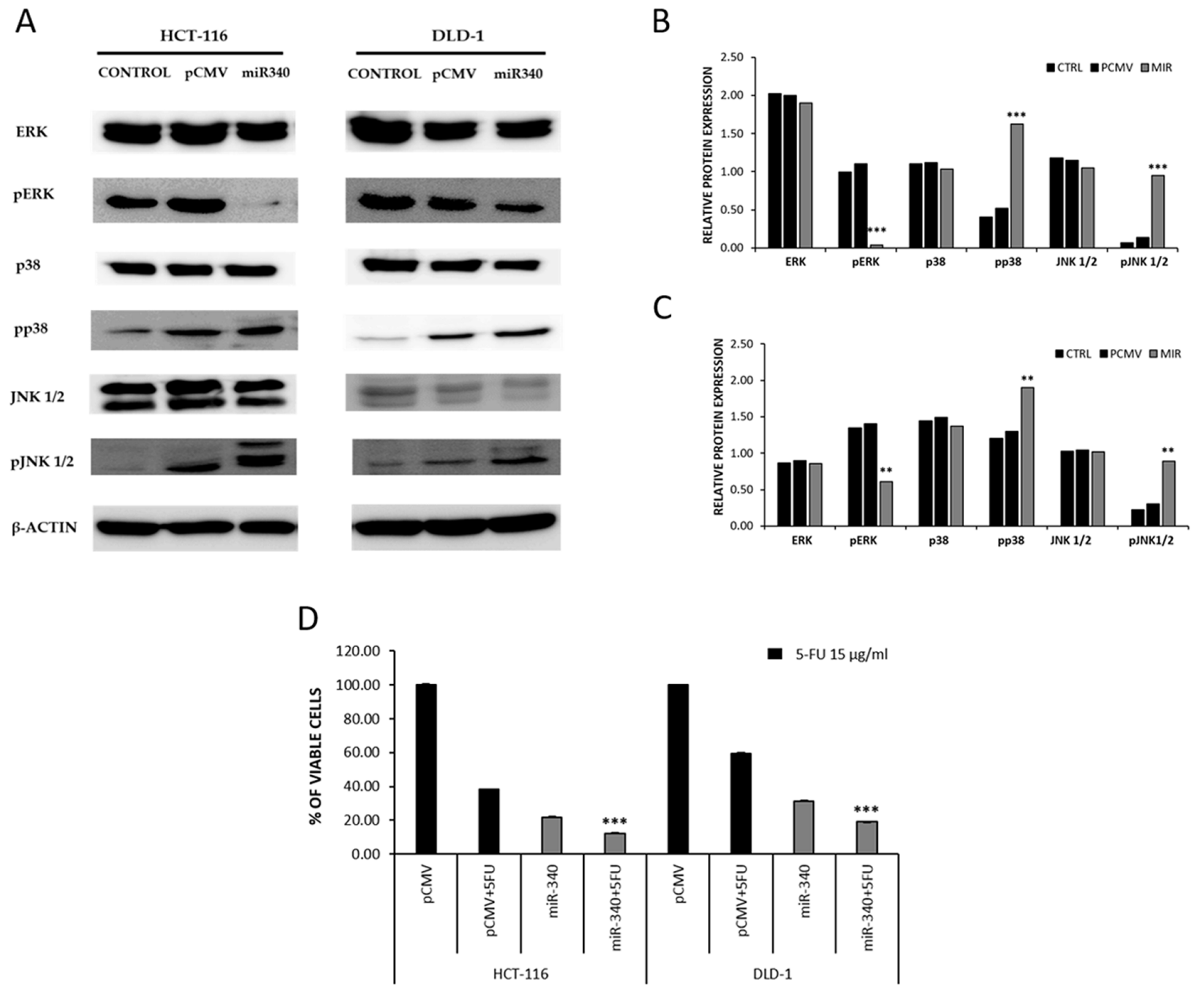
miR-340 regulates colon cancer via MAPK pathway and downregulation of REV3L sensitizes cancer cells to 5-Flurouracil *in vitro* **(A)** Cells were transfected with pCMV, pCMV-miR-340 plasmids or not transfected (control); 48 h after transfection, protein samples were used to perform western blot analysis. The downstream targets of MAPK pathway total ERK, p-ERK, total p38, p-p38, total JNK 1/2, p-JNK 1/2, was analyzed. β-Actin was used as a loading control. **(B, C)** The relative protein expressions of the control, pCMV and pCMV-miR-340 transfected HCT-116 and DLD-1 colon cancer cells were compared and represented as graph. **(D)** The sensitization of miR-340 treated HCT-116 and DLD-1 colon cancer cells to 5-FU was analyzed by cell proliferation MTS assay. HCT-116 and DLD-1 colon cancer cells were transfected with pCMV and pCMV-miR-340 plasmids followed by 5-FU treatment at concentration of 15μg/ml and incubated for 48 h. The cell viability in pCMV-miR-340 and 5-FU treated cells was compared to pCMV and 5-FU treated cells. The data are presented as means with SDs for three independent experiments. ^*^p<0.05; ^**^p<0.01; ^***^p<0.001.

To further understand the mechanism whereby miR-340 targets REV3L and controls cancer progression, we analyzed the MAPK pathway using western blot. HCT-116 and DLD-1 colon cancer cells were transfected with pCMV and/or pCMV-miR-340 plasmids, and then the downstream targets of MAPK specific for proliferation (ERK) and apoptosis (JNK and p38) were analyzed from the protein samples. Western blot results showed no significant differences in ERK, JNK, and p38 levels, whereas the phosphorylated ERK (p-ERK) levels were significantly lower in miR-340 treated HCT-116 and DLD-1 cells. On the other hand, p-JNK and p-38 (markers of apoptosis) levels were higher in miR-340 transfected cells (Figure 7A, 7B). These results suggest that miR-340 regulates colon cancer cell progression and apoptosis via the MAPK pathway.

### miR-340 downregulated markers of metastasis and enhanced the cytotoxicity of 5-FU in colon cancer cells

To examine further the impact of miR-340 on colon cancer cell metastasis. HCT-116 and DLD-1 colon cancer cells were transfected with pCMV and/or pCMV-miR-340 plasmids and RNA samples were collected. qPCR was performed to evaluate the mRNA expressions of the common markers of metastasis (MMP-2 and MMP-9). miR-340 treated colon cancer cells expressed significantly less MMP-2 and MMP-9 than pCMV treated cells (Figure 6C, 6D). These results suggest that miR-340 also plays important roles in the control the metastatic property of colon cancer cells.

Several reports have emphasized the role played by REV3L in inducing drug resistance to cancer cells. To explore this phenomenon, we examined the cytotoxicity of 5-FU, a drug commonly used in treatment for colon cancer, in miR-340 treated colon cancer cells. HCT-116 and DLD-1 colon cancer cells were transfected with pCMV and/or pCMV-miR-340 plasmids, and REV3L+miR-340 plasmids. The cells were further treated with 5-FU at a concentration of 15μg/ml and incubated for 48 h. MTS assays were then performed to evaluate cell viabilities. The results showed that co-treatment with miR-340+5-FU drastically reduced viable cell numbers as compared with pCMV+5-FU treatment. Cells treated with REV3L+miR-340+5-FU showed reduced cytotoxicity as compared to only miR-340+5-FU treated cells (Figure 7C).

### miR-340 inhibitor effect on tumor growth in an *in vivo* nude mouse model

**Figure 8:**
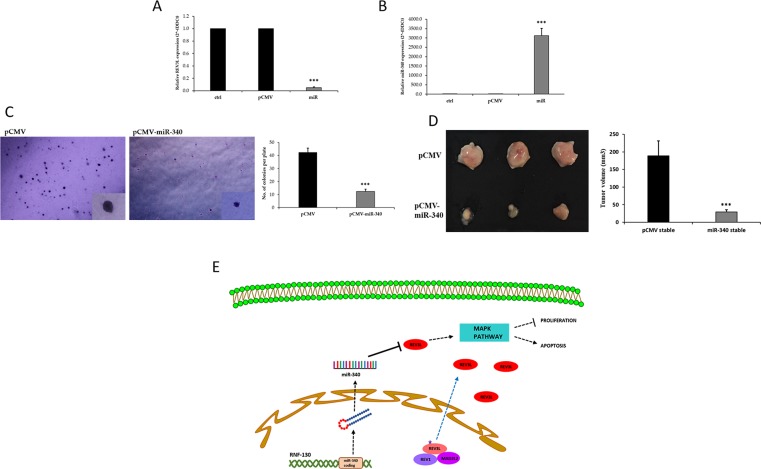
miR-340 inhibits tumorigenicity of colon cancer cells *in vivo* **(A)** Control and miR-340 overexpressing stable HCT-116 colon cancer cell line was made by transfecting pCMV and pCMV-miR-340 in HCT-116 colon cancer cells. 48 h after transfection, the cells were treated with desired concentrations of G418. mRNA expression of REV3L in not treated, pCMV and pCMV-miR-340 transfected HCT-116 cells selected with G418 was analyzed by qPCR. GAPDH gene was used as endogenous control. **(B)** The relative miR-340 expression level in pCMV-miR-340 transfected G418 selected HCT-116 stable colon cancer cell line compared to pCMV transfected G418 selected cells was analyzed by qPCR. U6 expression was used as endogenous control. **(C)** The number of colonies and the size of the colonies formed by the HCT-116-pCMV-miR-340 was less compared to HCT-116-pCMV stable cell line as analyzed by the soft agar colony formation assay. **(D)** The HCT-116-pCMV and HCT-pCMV-miR-340 stable colon cancer cells were subcutaneously injected into nude mice model (n=3). The tumor volume was calculated from the measured width and length. Graph represents smaller tumor volume in HCT-116-pCMV-miR-340 injected groups compared to HCT-116-pCMV injected groups. The data are presented as means with SDs for three independent experiments. ^*^p<0.05; ^**^p<0.01; ^***^p<0.001. **(E)** Schematic model of the REV3L location and interactions and the regulatory pathways involving miR-340 and REV3L in colon cancer. REV3L, a nuclear bound protein upon mutation breaks its known protein interactions and is mislocalized to the cytoplasm in colon cancer. miR-340 targets and downregulates REV3L thereby regulating the proliferation and apoptosis of colon cancer cells.

To determine the role of miR-340 in the tumorigenesis and progression of colon cancer and to explore the therapeutic potential of miR-340-based gene therapy, we assessed the effects of miR-340 expression in a nude mouse xenograft model using HCT-116-pCMV and HCT-116-pCMV-miR-340 overexpressing stable cell lines. Transfected cells were treated with G418 (800μg/ml) for two weeks, the concentration of G418 was gradually tapered (600μg/ml-300μg/ml) for following two weeks, and then cells were maintained at a G418 concentration of 300μg/ml thereafter. qPCR was then used to determine REV3L and miR-340 expressions. The results showed REV3L mRNA expression was higher and miR-340 expression was lower in HCT-116-pCMV-miR-340 stable cells than in HCT-116-pCMV stable cells (Figure 8A, 8B). Proliferation rates of the stablecells were investigated using a clonogenic soft agar assay. After 21 days of incubation, the colony numbers and sizes were smaller for HCT-116-miR-340 cells than for HCT-116-pCMV cells (Figure 8C). Two weeks after introducing stable HCT-116-pCMV and HCT-116-pCMV-miR-340 cells subcutaneously into the flanks of nude mice, tumor size measurements revealed tumor were smaller in the HCT-116-pCMV-miR-340 group (Figure 8D). Taken together, our results suggest miR-340 downregulated REV3L, inhibited colon cancer growth, and significantly inhibited tumor cell proliferation.

## DISCUSSION

The study was performed to determine how changes in the location and interactions of REV3L regulate colon cancer progression. Protein mislocalizations are being increasingly viewed as important aspects of several diseases including cancer, as mislocalized proteins display different biological functions that may facilitate disease progression [41, 42]. Many investigators have reported on the oncogenic role of REV3L in several types of cancers. Several reports have described consistent mutations in the REV3L gene in different cancer types, including, lung cancer, breast cancer, osteosarcoma, and head and neck cancer, and these mutations have been shown to confer tumorigenic properties [9, 11–13]. In the present study, we report for the first time mislocalization of the nuclear bound DNA mismatch repair protein REV3L to cytoplasm in colon cancer cells as evidence by immunostaining results. The mislocalization of REV3L was found to result in disruptions of its interactions with its protein partners REV1 and MAD2L2, as shown by PLA assay. These three proteins compose DNA Polymerase Zeta complex, which is responsible for mismatch repair during DNA replication. Furthermore, mislocalization of REV3L to cytoplasm might result in novel interactions and activate another cancer-related molecular pathways. On the other hand, mislocalization is likely to lead to the accumulation of DNA mutations and increase the risk of cellular neoplasticity. Our research team has previously reported on the impact of such mislocalizations on interactions in glioblastoma and stem cells [42].

We also observed that the expression of miR-340, a microRNA targeting REV3L, was suppressed in colon cancer. Several mutations in miR binding sites on the 3’-UTR region of REV3L have been previously reported, and these mutations prevent many tumor suppressing microRNAs binding to and downregulating REV3L [28]. In the present study, the miR-340 binding site on the 3’-UTR region of REV3L was sequenced and no mutations were found. Our reporter assay results confirmed prediction algorithm results that miR-340 is a good candidate for binding to REV3L. Furthermore, miR-340 expression was significantly lower in the two colon cancer cells than in normal colon cells marking, which also suggested a tumor suppressing role.

It was also observed the overexpression of miR-340 in colon cancer cells reduced proliferation and induced apoptosis, and the number of viable cells was dramatically lower in miR-340 treated HCT-116 and DLD-1 colon cancer cells than in the non-treated. Furthermore, treatment of colon cancer cells with miR-340 increase TUNEL positivity, and miR-340 was observed to control colon cancer cell proliferation by colony formation assays, which showed colony numbers were significantly reduced in miR-340 treated cells control. In addition, the wound healing assay showed miR-340 inhibited the migration of colon cancer cells.

We also expanded our study on the molecular mechanism whereby miR-340 controls colon cancer progression. Several groups have reported downregulation of REV3L induces p53 mediated apoptosis in different cancer types [3, 24, 43]. Here, we report for the first time, miR-340 mediated REV3L downregulation inhibits colon cancer cell proliferation and induces apoptosis via the MAPK pathway. Furthermore, while total ERK, JNK1/2, and p38 expressionswere similar in control and miR-340 treated colon cancercells, the phospho forms of these proteins differed in control and miR-340 treated cells. More specifically, pERK (a proliferation marker) expressionwas lower in miR-340 colon cancer cells than in control cells, and pJNK1/2 and pp38 levels were considerably higher in miR-340 colon cancer cells than in controls. These results confirm involvement of MAPK pathway regulation by miR-340 in colon cancer cell death. We also observed that the expressions of common metastasis markers (MMP-2 and MMP-9) were significantly lower in miR-340 treated colon cancer cells than in control cells. These results suggest miR-340 might be useful for controlling colon cancer metastasis.

The involvement of REV3L in drug resistance to several chemotherapeutic agents have been reported in different cancer types [18, 27]. In the present study, miR-340 based co-treatment with 5-Flurouracil enhanced treatment cytotoxic efficiency against colon cancer cells, that is, co-treatment with miR-340 and 5-FU increased cell death significantlyversus miR-340 or 5-FU treated cancer cells, which suggest combinations of miR-340 with chemotherapeutics might enhance therapeutic efficacy. However, co-treatment of REV3L+miR-340+5-FU reduced the cytotoxicity of 5-FU suggesting that REV3L confers drug resistance to colon cancer cells.

Furthermore, miR-340 overexpressing HCT-116 colon cancer cells formed smaller tumors than pCMV overexpressing HCT-116 cells in our nude mouse model, indicating REV3L downregulation by miR-340 inhibited colon cancer tumor growth and progression in our xenograft model.

In conclusion, the results obtained demonstrate the location and interactions of REV3L are quite different in colon cancer. It addition, it shows that miR-340, which is down-regulated in colon cancer, targets and downregulates REV3L, and regulates cancer cells apoptosis and proliferation *in vitro* and *in vivo*. Accordingly, our results suggest REV3L mislocation plays an important role in development of colon cancer and that the downregulation of REV3L by miR-340 controls proliferation and induces apoptosis (Figure 8E). These observations indicate miR-340 should be viewed as a potential means of treating colon cancer.

## MATERIALS AND METHODS

### Prediction of changes in REV3L location and interactions

Known locations of human REV3L were obtained from GO Cellular Component annotations using the AmiGO tool (http://amigo.geneontology.org/cgi-bin/amigo/go.cgi) and subcellular location prediction tools (including EXP, IDA, IPI, IMP, IGI, and IEP) considering only experimental evidence. Protein translocation predictions wereperformed using our previously reported conditional location predictor (CoLP) (Lee et al. 2013). To obtain the protein–protein interaction network, interactions were downloaded from the HPRD (Keshava Prasad et al. 2009), STRING, BIND (Bader et al. 2001), REACTOME (Joshi-Tope et al. 2005), and DIP (Salwinski et al. 2004) databases. In addition, interactions mined from prior literature were also included.

### microRNA prediction

hsa-miR-340 targeting the 3´UTR region of REV3L was predicted using microRNA.org-Targets and Expressions, miRDB, miRBASE, miRANDA, and TargetScan.

### Cell culture

A normal colon cell line (CCD-18Co) and two colon cancer cell lines (HCT-116, DLD-1) were purchased from the American Type Cell Culture collection. Cells were maintained in RPMI 1640 medium (HyClone) supplemented with 10% fetal bovine serum (Gibco), and 1% penicillin and streptomycin (Gibco) at 37°C in 5% CO_2_/95% air humidified atmosphere.

### Transfections

The precursor form of hsa-miR-340 (Thermo Scientific Co Inc., # C-301081-01-0010) or positive control miR (Thermo Scientific, #CP-004000-01-20) was overexpressed in the HCT-116 and DLD-1 cancer cell lines using Oligofectamine reagent (Invitrogen). In certain experiments and generation of stable clones, expression plasmid for human microRNA miR-340 (OriGene Tech., #SC400352) or control pCMV plasmids were transfected using Lipofectamine LTX and Plus reagent (Invitrogen).

### Generation of stable cell line

Briefly, HCT-116 cells were transfected with pCMV or pCMV-miR-340 plasmids for 48h, and then treated with 600μg/ml of G418 for two weeks and 400μg/ml of G418 for the following two weeks. Gradually the G418 concentration reduced to 300μg/ml, and thereafter, at this G418 concentration was maintained. The selected cells were pCMV or pCMV-miR-340 overexpressing.

### Immunofluorescence staining

Normal colon cell line (CCD-18Co) and two colon cancer cell lines (HCT-116, DLD-1) were prepared in 8-well chamber slides, rinsed in phosphate buffered saline (PBS, pH=7.4), fixed in 100% methanol for 20 mins, and washing three times with PBS. Non-specific antibody binding was blocked using normal serum (Vector laboratories) for 1 hour at room temperature. Cells were then incubated overnight with protein-specific antibodies at 4°C, washed with PBS, and incubated with secondary antibody at room temperature for 1 hour (Supplementary Table 1). Nuclei were counterstained with DAPI (4’6-diamino-2-phenilindole; 1 μg/ml, Sigma-Aldrich) for 20 secs, and after washing cells with PBS, they were mounted on glass slides using VECTASHIELD mounting media (Vector Laboratories), cover slipped, and analyzed using an LSM 710 confocal microscope (Carl Zeiss).

### Proximity ligation assay

CCD-18Co, HCT-116 and DLD-1 cells were prepared in 8-well chamber slides and allowed to attach for 24 hours. Cells were then rinsed in PBS, fixed in 100% methanol for 20 min, washed three times with PBS, and incubated overnight with protein-specific antibodies at 4°C. Proximity ligation assays were performed using the Duolink InSitu PLA Assay Kit (Sigma-Aldrich), and nuclei were DAPI stained. Specimens were mounted using Vectashield mounting media and analyzed under a LSM 710 confocal microscope (Carl Zeiss). Numbers of *in situ* PLA signals per cell were counted by semiautomatic image analysis using Blob Finder V3.0.

### Real-time quantitative polymerase chain reaction (RT-qPCR)

Total RNAs were isolated from CCD-18Co cells, HCT-116 and DLD-1 colon cancer cell lines, and from control and miR-340 transfected HCT-116 or DLD-1 cells lines using an RNeasy Mini Kit (Qiagen), and then reverse transcribed into complementary DNAs (cDNAs) using the PrimeScript First Strand cDNA Synthesis Kit (Takara). RT-qPCR reactions were run in a 20-μl mixture consisting of SYBR Premix Ex Taq (TaKaRa), cDNA template, and appropriate primers (Supplementary Table 2).

### REV3L 3´UTR mutation analysis

Genomic DNA was collected from CCD-18Co, HCT-116, and DLD-1 cells using the QIAamp DNA Mini Kit (Qiagen). 3´UTR region of REV3L was sequenced using the primers; for REV3L 3’UTR Fw 5’-ACCATATCTCCGGCAGTTATTAGA-3’ and Rev 3L 3´UTR Rv 5’-AAAACTCAGAAAAGGGTAGGGTAAG-3’ (Bioneer) and the mutations in the hsa-miR-340 binding region were analyzed by sequencing.

### Luciferase reporter assay

REV3L 3´UTR was created and cloned to firefly luciferase-expressing vector psi-CHECK2 for the luciferase assay. HCT-116 and DLD-1 cells were seeded in 6-well plates at 1×10^5^ cells/well the day before transfection. Cells were co-transfected with the psiCHECK2-REV3L-3´UTR and pCMV or pCMV-miR-340, psiCHECK2-REV3L-3´UTR-Deletion and pCMV or pCMV-miR-340 using Lipofectamine LTX and Plus reagent (Invitrogen). After 48h of incubation, luciferase activities were determined using the Dual-Luciferase Reporter System (Promega).

### Cell viability assay

Cell activity was determined using the Ez-Cytox Cell Viability Assay; a MTS (3-(4,5-dimethylthiazol-2-yl)-5-(3-carboxymethoxyphenyl)-2-(4-sulfophenyl)-2H-tetrazolium, inner salt) based assay. HCT-116 and DLD-1 cells were seeded uniformly in 96-well plates at a density of 1×10^4^ cells/ml and left overnight to attach. Cells were then transfected with the control microRNA and miR-340 mimics using Oligofectamine (Invitrogen) and incubated for 48 hours. MTS assay was performed according to the manufacturer's instructions. The staining intensities in culture medium (proportional to live cell numbers) are presented as spectrophotometry determined absorbances obtained at 450 nm.

### TUNEL staining

HCT-116 or DLD-1 cells were seeded at 1×10^5^ in six well plates, and 24 h after seeding, were transfected with pCMV and pCMV-miR-340 plasmid and incubated for 48 h. TUNEL reaction mixture (Roche) was then added, and samples were incubated for 60 min at 37°C in a humidified atmosphere in the dark. Slides were rinsed 3 times in PBS, and cells were mounted on glass slides using VECTASHIELD mounting media (Vector laboratories), cover slipped, and analyzed under a LSM 710 confocal microscope (Carl Zeiss). The numbers of TUNEL positive cells werecounted and plotted on a graph.

### Colony formation assay

Briefly, HCT-116 or DLD-1 cells were transfected with pCMV and pCMV-miR-340 plasmid and incubated for 48h. Cells were then trypsinized, seeded (500 cells/well) in 6-well plates and cultured for two weeks; medium was refreshed every 2 days. Cells were then fixed with methanol (15 min) and stained with 0.1% crystal violet. Colonies containing >50 cells were counted and plotted on a graph.

### Wound healing assay

HCT-116 or DLD-1 cells were transfected with pCMV or pCMV-miR-340 plasmid and incubated for 48h, trypsinized, and seeded (∼1× 10^6^ cells) in the wells of 6-wells plate and until monolayers formed. These cell layers were then scratched with a sterile Eppendorf tip (Sigma), washed 3 times with PBS, and incubated in fresh serum-free RPMI-1640 medium for 48h. Numbers of migrating cells observed 24 h and 48 h after scratching were counted under an inverted microscope (Olympus) and photographed. Numbers of migrated cells were calculated using GraphPad Prism 5 and plotted on a graph.

### Western blot analysis

HCT-116 or DLD-1 cells were seeded at a density of 1×10^5^ in 6-well plates, and 24h later were transfected with pCMV and pCMV-miR-340 plasmids and incubated for 48 h. Cell lysates were prepared using the EzRIPA Lysis kit (ATTO Corp), containing protease and phosphatase inhibitors, sonicated, and centrifuged at 14,000 X g for 20 mins at 4°C. Total protein concentrations were measured using QUBIT Fluorometric Quantitation method (Life Technologies) according to the manufacturer's instructions. Equal amounts (50 μg) of proteins were separated in 12% polyacrylamide gels and transferred to nitrocellulose membranes (Millipore). Proteins were detected with protein specific antibodies, and ECL (Sigma-Aldrich) detection reagent was used to visualize immunoreactive proteins on membranes.

### Soft agar colony formation assay

For the soft agar colony formation assay, a bottom layer of agar (0.5%) mixed with RPMI-1640 supplemented with 10% FBS was poured first. After solidification, 0.3% agar mixed with RPMI-1640 supplemented with 10% FBS and HCT-116 pCMV or pCMV-miR-340 stable cells (5×10^3^) was poured on top and allowed to cool and incubated at 37°C for 21 days. The culture media was changed once or twice weekly. Colonies were visualized by staining with 0.005% crystal violet and counted.

### 5-Fluorouracil co-treatment

HCT-116 or DLD-1 cells were seeded uniformly in 96-well plates at a density of 1×10^4^ cells/ml and left overnight to attach. Cells were transfected with pCMV and/or pCMV-miR-340 plasmids using Lipofectamine (Invitrogen) and then treated with 5-FU (15μg/ml). MTS (Ez-Cytox Cell Viability Assay) was performed according to the manufacturer's protocol. Intensity of staining in culture medium as determined by spectrophotometry at 450 nm was used as a surrogate of live cell numbers.

### The nude mouse model

Female BALB/c nude mice (aged 6–8 weeks) were obtained from Orient Bio Inc, Korea. HCT-116-pCMV overexpressing and HCT-116-hsa-miR-340 overexpressing stable cells (2×10^6^ cells/tumor) were mixed in 100 ml PBS and subcutaneously injected into the mice. Tumors were allowed to develop for about two weeks and tumor volumes were measured using an external caliper and the formula:

$$\text{Tumor volume}=\frac{\text{Length}\times {\text{Width}}^{2}}{2}$$

### Statistical analysis

Results are presented as means ± standard deviations (SDs). Statistical significance was evaluated using the Student's t-test and P values of < 0.05 were considered significant.

## SUPPLEMENTARY MATERIALS FIGURE AND TABLES


