# Supplementary material for: MicroRNA-340 inhibits the proliferation and promotes the apoptosis of colon cancer cells by modulating REV3L
Source: Oncotarget. 2017 Dec 26;9(4):5155–68. doi: 10.18632/oncotarget.23703 (PMC5797040; doi:10.18632/oncotarget.23703)
Supplement: Supplementary file 1 [file oncotarget-09-5155-s001.pdf]

MicroRNA-340 inhibits the proliferation and promotes the apoptosis of colon cancer cells by modulating REV3L

SUPPLEMENTARY MATERIALS

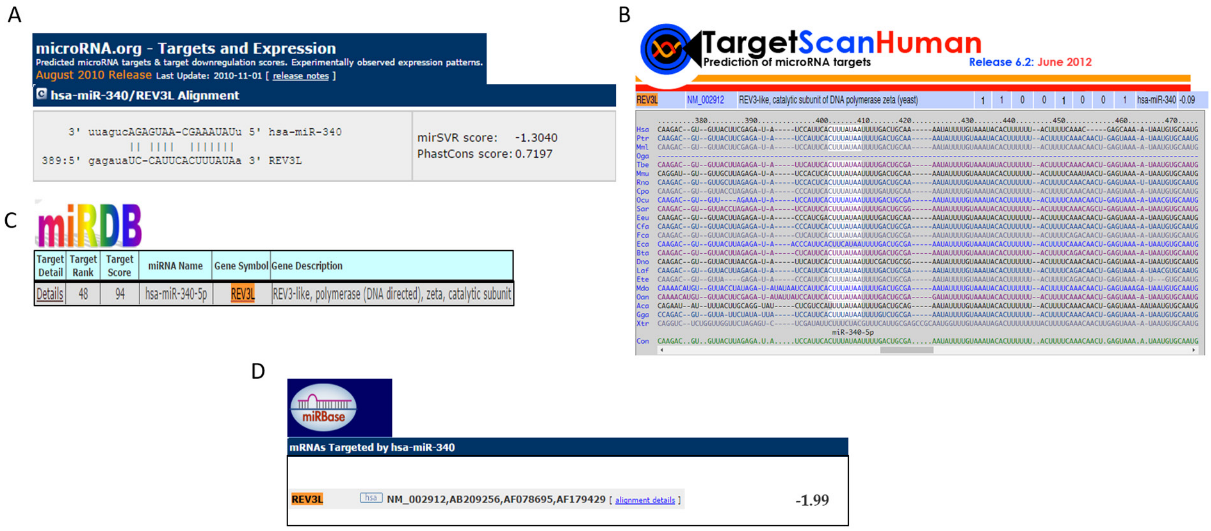

Supplementary Figure 1: microRNA prediction tools. The microRNA targeting the 3'UTR of REV3L was predicted using general microRNA prediction tools. **(A)** microRNA.org. **(B)** TargetScan. **(C)** miR Data Base. **(D)** miRBASE.

Supplementary Table 1: List of antibodies used for immunostaining and western blot

	Host	Company (Cat No)	Application	
			IHC	WB
REV3L	Goat	Santa Cruz (sc-5939)	1:100	-
REV1	Goat	Acris (AP31943PU-N)	1:100	-
MAD2L2	Rabbit	Biorbytb(Orb100056)	1:100	-
SAPK/JNK	Rabbit	Cellsignaling (#9252S)	-	1:1000
pSAPK/pJNK	Rabbit	Cellsignaling (#9251S)	-	1:1000
ERK 1/2	Rabbit	Zymed (13-6200)	-	1:1000
pERK 1/2	Rabbit	Abcam (ab8227)	-	1:1000
p38	Rabbit	Cellsignaling (#9212S)	-	1:1000
pp38	Rabbit	Cellsignaling (#9211S)	-	1:1000
GAPDH	Mouse	Millipore (AB2302)	-	1:1000
Fibrillin	Rabbit	Cell Signaling (#2639)	-	1:1000
Peroxidase anti-mouse	Mouse	Vector (PI2000)	-	1:10000
Peroxidase anti-rabbit	Rabbit	Vector (PI 1000)	-	1:10000
Alexa Fluor 488	Goat	Invitrogen (A11055)	1:500	-

Supplementary Table 2: List of primers used for real-time quantitative polymerase chain reaction and Luciferase reporter assay

Gene	Forward primer (5' – 3')	Reverse primer (5' – 3')
hGAPDH	GGAGCGAGATCCCTCCAAAAT	GGCTGTTGTCATACTTCTCATGG
REV3L	TGATGTCTTCAGCTGGTAT	CCGCCCTTCAGGTTCACTT
hsa-miR-340-5p- Stem loop	GTCGTATCCAGTGCAGGGTCCGAG GTATTCGCACTGGATACGACAATCAG	-
hsa-miR-340-5p	GCGGTTATAAAGCAATGAGA	-
RNU6 Stem loop	GTCGTATCCAGTGCAGGGTCCGAGGTA TTCGCACTGGATACGACTATGGAAC	-
RNU6	CTGCGCAAGGATGACACGCA	-
Universal reverse primer	-	GTGCAGGGTCCGAGGT
RNF130	GCTCACGTTTCGCATCGAC	AACCGGGTTTGTGGATCACAG
MMP 2	ATAACCTGGATGCCGTCGT	AGGCACCCTTGAAGAAGTAGC
MMP 9	GAACCAATCTCACCGACAGG	GCCACCCGAGTGTAACCATA
3'UTR- REV3L	TAGGCGATCGCTCGAGACCATAT CTCCGGCAGTTATTAGA	TTGCGGCCAGCGGCCGCAAAA CTCAGAAAAGGGTAGGGTAAG
3'UTR Del- REV3L	TAAGCAGAATTCCAAACGAGCA AAATAATGTGC	TAAGCAGAATTCGAAGTAACAC GTCTTGATAC
